# Graminex Pollen: Phenolic Pattern, Colorimetric Analysis and Protective Effects in Immortalized Prostate Cells (PC3) and Rat Prostate Challenged with LPS

**DOI:** 10.3390/molecules23051145

**Published:** 2018-05-11

**Authors:** Marcello Locatelli, Nicola Macchione, Claudio Ferrante, Annalisa Chiavaroli, Lucia Recinella, Simone Carradori, Gokhan Zengin, Stefania Cesa, Lidia Leporini, Sheila Leone, Luigi Brunetti, Luigi Menghini, Giustino Orlando

**Affiliations:** 1 Department of Pharmacy, G. D’Annunzio University of Chieti-Pescara, 66100 Chieti, Italy; marcello.locatelli@unich.it (M.L.); claudio.ferrante@unich.it (C.F.); annalisa.chiavaroli@unich.it (A.C.); lucia.recinella@unich.it (L.R.); l.leporini@unich.it (L.L.); sheila.leone@unich.it (S.L.); luigi.brunetti@unich.it (L.B.); luigi.menghini@unich.it (L.M.); giustino.orlando@unich.it (G.O.); 2 Department of Urology, University of Milan, ASST Santi Paolo e Carlo, 20142 Milan, Italy; nclmacchione@gmail.com; 3 Department of Biology, Science Faculty, Selcuk University, Konya 42075, Turkey; gokhanzengin@selcuk.edu.tr; 4 Dipartimento di Chimica e Tecnologie del Farmaco, Sapienza University of Rome, 00185 Rome, Italy; stefania.cesa@uniroma1.it

**Keywords:** pollen, PGE_2_, NFκB mRNA, phenolic pattern, colorimetric analysis, inflammation

## Abstract

Prostatitis, a general term describing prostate inflammation, is a common disease that could be sustained by bacterial or non-bacterial infectious agents. The efficacy of herbal extracts with antioxidant and anti-inflammatory effects for blunting the burden of inflammation and oxidative stress, with possible improvements in clinical symptoms, is under investigation. Pollen extracts have been previously reported as promising agents in managing clinical symptoms related to prostatitis. The aim of the present work was to evaluate the protective effects of Graminex pollen (Graminex^TM^, Deshler, OH, USA), a commercially available product based on standardized pollen extracts, in rat prostate specimens, ex vivo. In this context, we studied the putative mechanism of action of pollen on multiple inflammatory pathways, including the reduction of prostaglandin E_2_ (PGE_2_), nuclear factor kappa-light-chain-enhancer of activated B cells (NFκB), and malondialdehyde (MDA), whose activities were significantly increased by inflammatory stimuli. We characterized by means of chromatographic and colorimetric studies the composition of Graminex pollen to better correlate the activity of pollen on immortalized prostate cells (PC3), and in rat prostate specimens challenged with *Escherichia coli* lipopolysaccharide (LPS). We found that Graminex pollen was able to reduce radical oxygen species (ROS) production by PC3 cells and MDA, NFκB mRNA, and PGE_2_ levels, in rat prostate specimens. According to our experimental evidence, Graminex pollen appears to be a promising natural product for the management of the inflammatory components in the prostate.

## 1. Introduction

Prostatitis, a general term describing prostate inflammation, is a common disease that could be sustained by bacterial or non-bacterial infectious agents [1]. Considering the inflammatory pathways involved in prostatitis, the efficacy of herbal extracts endowed with antioxidant and anti-inflammatory effects for reducing the burden of inflammation and oxidative stress, with possible improvements in clinical symptoms [2,3], is under investigation. Pollen extract has been previously reported as a promising natural product in managing clinical symptoms related to prostatitis [4]. Its efficacy was comparable with that of the anti-inflammatory drug ibuprofen, and has been related, albeit partially, to the reduced activity of pro-inflammatory interleukin (IL)-8 [5]. This is consistent with the reported anti-inflammatory effects related to pollen in multiple experimental models of inflammation and oxidative stress [4].

Pollen was reported to be characterized by a large plethora of substances, such as complex mixtures of carbohydrates, proteins, amino acids, phenolic compounds, minerals, vitamins, lipids, and organic acids [6]. This valuable natural product might be further analyzed in terms of its phenolic pattern, due to the recognized diversity of its mixed botanical origin, seasonal variation, and organoleptic properties. For example, specific secondary metabolites were proposed as analytical/floral markers for the palynological analysis of pollen. Furthermore, according to storage conditions and traditional consumption, bee pollen could undergo enzymatic and thermal degradation. Although analytical high performance liquid chromatography (HPLC-PDA) detection and quantitation of specific phenolics is a wide and routine analytical approach for characterizing the quali-quantitative composition, no data in the literature shed light on the colorimetric parameters influencing the pollen color and the means by which they change under stressing working conditions (e.g., artificial heating at high temperature) [7].

In this context, the aims of the present work were to characterize, by means of chromatographic and colorimetric studies, the composition of Graminex pollen (Graminex^TM^) as a mixture of standardized pollen of rye grass (*Secale cereale* L.), corn (*Zea mays* L.), and timothy (*Phleum pratense* L.), beyond the reported data specified by the producer. This was undertaken in order to evaluate the antiproliferative activity of pollen on immortalized prostate cells (PC3), and the protective effects in an experimental model of tissue inflammation constituted by ex vivo prostate specimens challenged with *Escherichia coli* lipopolysaccharide (LPS). In addition, we studied the putative mechanism of action of pollen on multiple inflammatory pathways, including the impact on prostaglandin E_2_ (PGE_2_) and nuclear factor kappa-light-chain-enhancer of activated B cells (NFκB), whose activities were significantly increased by inflammatory stimuli, including LPS [8,9]. Finally, we investigated the protective ability of pollen regarding radical oxygen species (ROS) production, the levels of malondialdehyde (MDA), a well-established marker of lipid peroxidation [10,11], as well as the antioxidant activity and chelating properties. This evidence justifies a proper recommendation of the tested Graminex pollen as a novel protective product against prostate inflammation. To further corroborate the biological potential of this complex natural product, we also evaluated the inhibitory activity against a panel of target enzymes (cholinesterases, tyrosinase, α-amylase, and α-glucosidase) that are important for other diseases, albeit diseases which are partially correlated to prostatitis.

## 2. Material and Methods

### 2.1. Chemicals and Standards

Flower Pollen 63/Graminex^TM^ G63 powder (α-amino acids not less than 3.6 mg/250 mg and phytosterols not less than 0.2 mg/250 mg) was kindly provided by Idipharma (Catania, Italy); the batch number was 1500-06-00-1; this product is commercially available on the market in Italy.

Chemical standards used for the validated HPLC procedure: catechin, gallic acid, 4-hydroxybenzoic and chlorogenic acid, epicatechin, vanillic and syringic acid, crocin, 3-hydroxybenzoic acid, 3-hydroxy-4-methoxybenzaldehyde, *p*-coumaric and sinapinic acid, rutin, *t*-ferulic acid, naringin, 2,3-dimethoxybenzoic acid, benzoic acid, *o*-coumaric acid, quercetin, harpagoside, *t*-cinnamic acid, naringenin, and carvacrol (>98%) were purchased from Sigma-Aldrich (Milan, Italy). Solvents such as acetonitrile (HPLC-grade), methanol (HPLC-grade), and acetic acid (≥99%) were obtained from Carlo Erba Reagents (Milan, Italy). Safranal (>88%) was purchased from Sigma-Aldrich (Milan, Italy) and further purified by column chromatography on silica gel (high purity grade, pore size 60 Å, 230–400 mesh particle size) using ethyl acetate:*n*-hexane (1:3) as the eluent. ^1^H and ^13^C NMR and IR spectra of the purified product were in agreement with those reported in the literature [12]. A centrifuge, model 5804 (Eppendorf, Hamburg, Germany), vortex (VELP Scientifica Srl, Usmate, Italy), and ultrasound bath (Falc Instruments, Treviglio, Italy) were used as additional equipment. All the other chemicals for in vitro assays were purchased from Sigma-Aldrich (Milan, Italy).

### 2.2. HPLC-PDA Analyses

A validated HPLC-PDA polyphenolic pattern was applied as previously reported [13] by means of HPLC Waters liquid chromatograph (model 600 solvent pump, 2996 PDA, Waters Spa, Milford, MA, USA), connected to a C18 reversed-phase column (Prodigy ODS-3, 4.6 × 150 mm, 5 µm; Phenomenex, Torrance, CA, USA). The mobile phase was directly *on-line* degassed by using a Biotech 4CH DEGASI Compact (Onsala, Sweden). Empower v.2 Software (Waters Spa, Milford, MA, USA) was used to collect and analyze data. All samples were accurately weighed and dissolved in the mobile phase, sonicated, centrifuged at 12000 g, and directly injected (20 µL) into HPLC-PDA system. For over range samples, 1:10 dilution factor was applied. Data are reported as mean ± standard deviation of three independent measurements.

### 2.3. Colorimetric Analysis

The pollen samples, as powder, were submitted to colorimetric analyses and monitored for colour modification during storage at 55 °C for prolonged periods. CIELAB (as defined by the “Commission Internationale de l’Eclairage”), parameters, namely L*, a*, b*, C*_ab_ and *h_ab_*, were measured using a colorimeter X-Rite SP-62 (X-Rite Europe GmbH, Regensdorf, Switzerland), equipped with a D65 illuminant, an observer angle of 10°, and an integration sphere, to determine the colour reflectance.

For the measurements, the powder sample was divided into four portions that were subject to analysis at *t*°, and then stored at 55 ± 1 °C in a closed recipient and analysed at *t* = 24, 48, 72, 144, 216, and 288 h. Each reported value is the median of ten measurements, performed randomly on the surface of the cell. Colour description is based on three parameters: L* defines the lightness, and varies between 0 (absolute black) and 100 (absolute white); a* measures the greenness (−a*) or the redness (+a*), and b* that measures the blueness (−b*) and the yellowness (+b*); C*_ab_ (chroma, saturation) expresses a measure of colour intensity, while *h_ab_* (hue, colour angle) is the attribute of appearance by which a colour is identified according to its resemblance to red, yellow, green, or blue, or to a combination of two of these attributes in sequence. Cylindrical coordinates C*_ab_ and *h_ab_* are calculated from the parameters a* and b* using the equations C*_ab_ = (a*^2^ + b*^2^)^½^ and *h_ab_* = tan^−1^ (b*/a*) [14].

### 2.4. In Vitro Studies

PC3 cells were cultured in DMEM (Dulbecco's Modified Eagle Medium, Euroclone, Pero, Italy) supplemented with 10% (*v*:*v*) heat-inactivated fetal bovine serum, and 1.2% (*v*:*v*) penicillin G/streptomycin, in a 75 cm^2^ tissue culture flask (*n* = 5 individual culture flasks for each condition). The cultured cells were maintained in a humidified incubator with 5% CO_2_ at 37 °C. For cell differentiation, PC3 cell suspensions at a density of 1 × 10^6^ cells/mL were treated with various concentrations (10, 50, and 100 ng/mL) of phorbol myristate acetate (PMA, Fluka, Milan, Italy) for 24 h or 48 h (induction phase). Thereafter, the PMA-treated cells were washed twice with an ice-cold pH 7.4 phosphate buffer solution (PBS) to remove PMA and non-adherent cells, whereas the adherent cells were further maintained for 48 h (recovery phase). The morphology of cells was examined under an inverted phase-contrast microscope [15]. To assess the basal cytotoxicity of the extracts, a preliminary viability assay was performed on 96 microwell plates, using 3-(4,5-dimethylthiazol-2-yl)-2,5-diphenyltetrazolium bromide (MTT) test. Cells were incubated with aqueous pollen extracts (ranging concentration 10–500 μg/mL) for 24 h. 10 μL of MTT solution (5 mg/mL) were added to each well and incubated for 3 h. The formazan dye formed was extracted with dimethyl sulfoxide, and absorbance recorded as previously described [16]. Effects on cell viability were evaluated in comparison to the untreated control group.

### 2.5. ROS Generation

ROS generation was assessed using a ROS-sensitive fluorescence indicator, 2′,7′-dichlorodihydrofluorescein diacetate (DCFH-DA). When DCFH-DA is introduced into viable cells, it penetrates the cell and is deacetylated by intracellular esterases, to form 2′,7′-dichlorodihydrofluorescein (DCFH), which can react quantitatively with ROS within the cell, and be converted to 2′,7′-dichlorofluorescein (DCF), which is detected by a fluorescence spectrophotometer. To determine intracellular effects on ROS production, cells were seeded in a black 96-well plate (1.5 × 10^4^ cells/well) in a medium containing scalar concentrations of aqueous pollen extracts. Immediately after seeding, the cells were stimulated for 1 h with H_2_O_2_ (1 mM). After incubation with DCFH-DA (20 μM) for 30 min, the fluorescence intensity was measured at an excitation wavelength of 485 nm, and an emission wavelength of 530 nm, using a fluorescence microplate reader [16].

### 2.6. Ex Vivo Studies

Male adult Sprague-Dawley rats (200–250 g) were housed in Plexiglas cages (40 cm × 25 cm × 15 cm), two rats per cage, in climatized colony rooms (22 ± 1 °C; 60% humidity), on a 12 h/12 h light/dark cycle (light phase: 07:00–19:00 h), with free access to tap water and food, 24 h/day throughout the study, with no fasting periods, as previously described [17]. Housing conditions and experimentation procedures were strictly in accordance with the European Union ethical regulations on the care of animals for scientific research. According to the recognized ethical principles of “Replacement, Refinement and Reduction of Animals in Research”, prostate specimens were obtained as residual material from vehicle-treated rats randomized in our previous experiments, approved by Local Ethical Committee (University “G. d’Annunzio” of Chieti-Pescara) and Italian Health Ministry (Project N. 880 definitely approved by Italian Health Ministry on 24 August 2015). Considering the physiological circadian rhythms of animals, rats were sacrificed at 9:00 a.m., as previously reported [18], and whole prostates (including all four lobes: ventral, dorsal, lateral, and anterior) were immediately collected and maintained in a humidified incubator with 5% CO_2_ at 37 °C for 4 h, and entirely submerged in 2 mL of an RPMI buffer with added bacterial LPS (10 µg/mL) (incubation period). During the incubation period, tissues were treated with the aqueous pollen extract (100 μg/mL). Tissue samples were collected, and PGE_2_ levels (ng/mg wet tissue) were measured by radioimmunoassay (RIA), as previously reported [19,20]. In addition, we evaluated the gene expression of NFκB following treatment, as previously reported [21,22]. Finally, the MDA level was determined by the thiobarbituric acid reactive substances (TBARS) method [23]. Briefly, tissue specimens were added with 1% H_3_PO_4_ and 0.6% thiobarbituric acid, and then incubated at 96 °C for 20 min. Finally, absorbance was recorded at 532 nm, and the MDA level was expressed as µg/mL.

### 2.7. Total Antioxidant Activity by Phosphomolybdenum Assay

The total antioxidant activity of pollen (dissolved in DMSO) was evaluated by the phosphomolybdenum method, according to Zengin et al. [24]. The sample solution (0.3 mL) was combined with a 3 mL of reagent solution (0.6 M sulphuric acid, 28 mM sodium phosphate and 4 mM ammonium molybdate), and the absorbance was recorded at 695 nm after 90 min incubation at 95 °C. Trolox was used as reference drug, and the results were expressed as trolox equivalents (mg TE/g) [13].

### 2.8. Radical Scavenging Activity

The radical scavenging effect of pollen (dissolved in DMSO) on the 1,1-diphenyl-2-picrylhydrazyl (DPPH) radical was estimated, according to Zengin et al. [24]. The sample solution in DMSO (1 mL) was added to 4 mL of a 0.004% solution of DPPH in methanol. The sample absorbance was recorded at 517 nm after 30 min incubation at room temperature in the dark.

The scavenging activity of pollen extract on the ABTS [2,2′-azino-bis(3-ethylbenzothiazoline)-6-sulphonic acid] radical cation was measured according to the method of Zengin et al. [13]. ABTS^+^ was produced directly by reacting a 7 mM ABTS solution with 2.45 mM potassium persulphate, and allowing the mixture to incubate for 12–16 h in the dark at room temperature. Prior to initiating the assay, the ABTS solution was diluted with methanol to an absorbance of 0.700 ± 0.02 at 734 nm. The sample solution in DMSO (1 mL) was added to ABTS^+^ solution (2 mL) and mixed, and the sample absorbance was recorded at 734 nm after 30 min incubation at room temperature. Trolox was used as reference drug, and the results were expressed as trolox equivalents (mg TE/g) [13].

### 2.9. Reducing Power (CUPRAC and FRAP Tests)

The cupric ion reducing activity (CUPRAC) was determined according to the method of Zengin et al. [13]. The sample solution in DMSO (0.5 mL) was added to a premixed reaction mixture containing CuCl_2_ (1 mL, 10 mM), neocuproine (1 mL, 7.5 mM) and NH_4_Ac buffer (1 mL, 1 M, pH 7.0). Similarly, a blank was prepared by adding the sample solution in DMSO (0.5 mL) to a premixed reaction mixture (3 mL) without CuCl_2_. The sample or blank absorbance was subsequently recorded at 450 nm after 30 min incubation at room temperature. The FRAP (ferric reducing antioxidant power) assay was carried out, as described by Zengin et al. [13]. The sample solution in DMSO (0.1 mL) was added to the premixed FRAP reagent (2 mL) containing an acetate buffer (0.3 M, pH 3.6), 2,4,6-tris(2-pyridyl)-*s*-triazine (10 mM) in 40 mM HCl, and ferric chloride (20 mM) in a ratio of 10:1:1 (*v*:*v*:*v*). The sample absorbance was subsequently recorded at 593 nm after 30 min incubation at room temperature. Trolox was used as reference drug, and the results were expressed as trolox equivalents (mg TE/g) [13].

### 2.10. Metal Chelating Activity on Ferrous Ions

Metal chelating activity on ferrous ions was evaluated by the method described by Zengin et al. [13]. Briefly, the sample solution in DMSO (2 mL) was added to FeCl_2_ solution (0.05 mL, 2 mM). The reaction was initiated by the addition of 5 mM ferrozine (0.2 mL). Similarly, a blank was prepared by adding a sample solution (2 mL) to FeCl_2_ solution (0.05 mL, 2 mM) and water (0.2 mL) without ferrozine. Then, the sample and blank absorbance were read at 562 nm after incubation for 10 min at room temperature. EDTA was used as a positive control and the results were expressed as EDTA equivalents (mg EDTAE/g) [13].

### 2.11. Enzyme Inhibition

#### 2.11.1. Cholinesterase Inhibition

The test solution (50 µL) was mixed with DTNB (5,5′-dithiobis(2-nitrobenzoic acid), 125 µL) and an enzyme (AChE or BChE) solution (25 µL) in Tris-HCl buffer (pH 8.0) in a 96-well microplate, and incubated for 15 min at room temperature. The reaction was then started with the addition of the corresponding substrates (acetylthiocholine iodide (ATCI) or butyrylthiocholine chloride (BTCl)) (25 µL). Similarly, a blank was prepared for each sample without the enzyme (AChE or BChE) solution. The absorbances of the sample and blank were registered at 405 nm after 10 min incubation at 25 °C. Results were given as milligrams of galantamine equivalents (GALAEs/g) [25].

#### 2.11.2. α-Amylase Inhibition

The test solution (25 µL) was mixed with α-amylase solution (50 µL) in a phosphate buffer (pH 6.9 with 6 mM sodium chloride) in a 96-well microplate, and incubated for 10 min at 37 °C. After this incubation, the reaction was started with the addition of starch solution (50 µL, 0.05%). Similarly, a blank was prepared for each sample (without the enzyme solution). The reaction mixture was incubated 10 min at 37 °C. The reaction was stopped with the addition of HCl (25 µL, 1 M), then the iodine-potassium iodide solution was added (100 µL). The absorbances of the sample and blank were evaluated at 630 nm. The absorbance of the blank was subtracted from that of the sample. Results were given as millimoles of acarbose equivalents (ACAEs/g) [26].

#### 2.11.3. α-Glucosidase Inhibition

Test solution (50 µL) was mixed with glutathione (50 µL), α-glucosidase solution (50 µL), in a phosphate buffer (pH 6.8) and PNPG (*p*-nitrophenyl-β-d-glucuronide, 50 µL) in a 96-well microplate, and incubated for 15 min at 37 °C. Similarly, a blank was prepared for each sample (without the enzyme solution). The reaction was stopped with sodium carbonate (50 µL, 0.2 M). The absorbances of the sample and blank were noted at 400 nm. Results were expressed as millimoles of acarbose equivalents (ACAEs/g) [24].

#### 2.11.4. Tyrosinase Inhibition

The test solution (25 µL) was mixed with a tyrosinase solution (40 µL) and phosphate buffer (100 µL, pH 6.8) in a 96-well microplate, and incubated for 15 min at 25 °C. The reaction was then started with L-DOPA (40 µL). Similarly, a blank was done for each sample (without containing the enzyme solution). The absorbances of the sample and blank were recorded at 492 nm after 10 min incubation at the room temperature. Results were expressed as milligrams of kojic acid equivalents (KAE/g) [24].

### 2.12. Statistical Analysis

Statistical analysis was performed using GraphPad Prism version 5.01 for Windows (GraphPad Software, San Diego, CA, USA). Means ± S.E.M. were determined for each experimental group and analyzed by one-way analysis of variance (ANOVA), followed by Newman-Keuls comparison multiple test. Statistical significance was set at *p* < 0.05. Regarding gene expression analysis, the comparative 2^−ΔΔCt^ method was used to quantify the relative abundance of mRNA, and then to determine the relative changes in individual gene expression (relative quantification) [27]. Finally, with regards to the animals that were randomized for each experimental group, the number was calculated on the basis of the “Resource Equation” [28], according to the guidelines suggested by the “National Centre for the Replacement, Refinement and Reduction of Animals in Research” (NC3RS).

## 3. Results and Discussion

**Figure 1:**
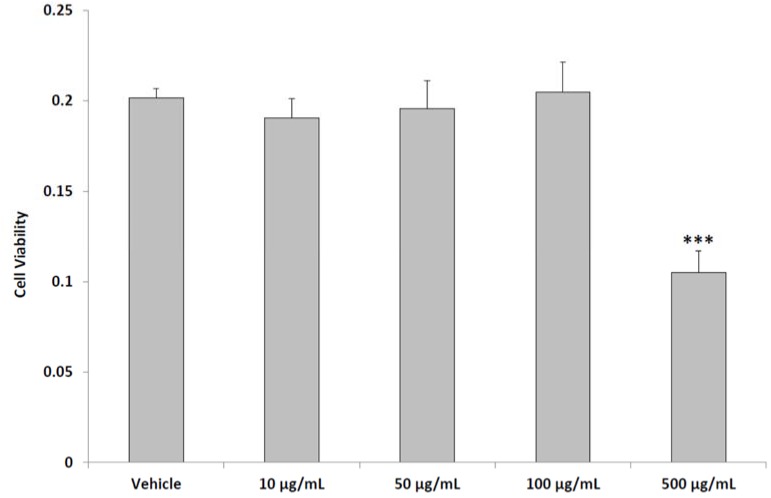
Effects of aqueous pollen extract (10–500 μg/mL) on PC3 cell line viability (ANOVA, *p* < 0.0001; post-hoc test *** *p* < 0.001 vs. vehicle group).

The MTT test revealed that the aqueous pollen extract was well tolerated by the PC3 cell line in the range 10–100 µg/mL, in agreement with the reported clinical studies by Cai et al. [4,5]. In contrast, at the highest tested concentration (500 µg/mL), we observed a reduction of cell viability related to the presence of the extract (Figure 1), thus supporting a possible anti-proliferative effect at high concentration.

**Figure 2:**
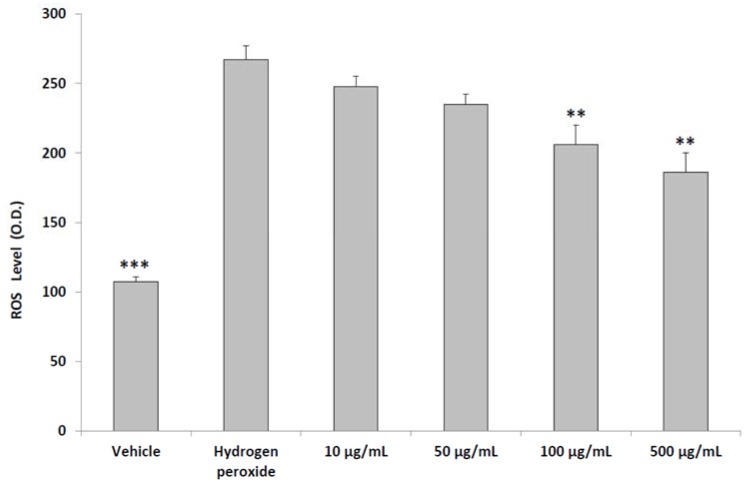
Effects of aqueous pollen extract (10–500 μg/mL) on ROS production from PC3 cell line (ANOVA, *p* < 0.0001; post-hoc test ** *p* < 0.01, *** *p* < 0.001 vs. hydrogen peroxide group).

The inhibitory effects on cell viability could be related to the capability of steroid fraction of pollen to induce PC3 cell apoptosis [29]. On the other hand, the aqueous pollen extract did not modify the viability of intestinal Caco-2, spermatogonial stem cells and sertoli cell lines [30,31]. In PC3 cells, we also observed a protective effect induced by the aqueous pollen extract (10–100 μg/mL) supplementation, which was able to inhibit hydrogen peroxide-induced ROS production (Figure 2).

Concerning this result, we performed a second set of experiments with the aim of elucidating the mechanism of the antioxidant role exerted by pollen, that emerged as a promising antioxidant agent in the employed biological models. In particular, we tested the in vitro antioxidant and chelating properties, finding that pollen displayed scavenging effects in phosphomolybdenum, ABTS, CUPRAC and FRAP tests better than Trolox, used as reference. In addition, we observed a discrete chelating ability (*p* < 0.05, Table 1).

**Figure 3:**
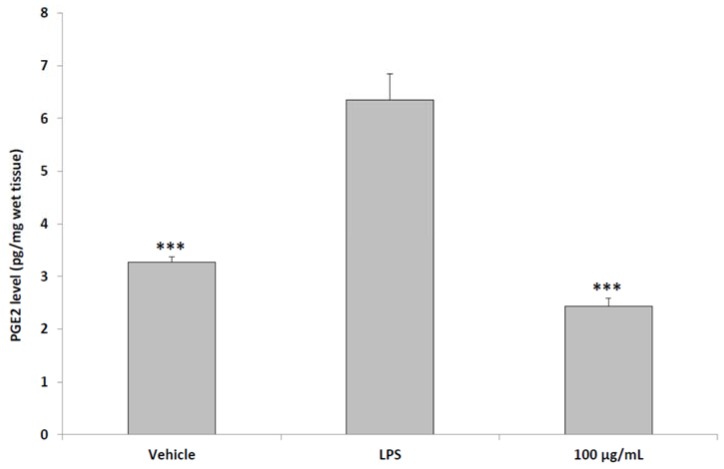
Effects of aqueous pollen extracts (100 μg/mL) on PGE_2_ production from isolated rat prostate specimens challenged with LPS (ANOVA, *p* < 0.0001; post-hoc test *** *p* < 0.001 vs. LPS group). Pollen extracts were given simultaneously with LPS.

**Figure 4:**
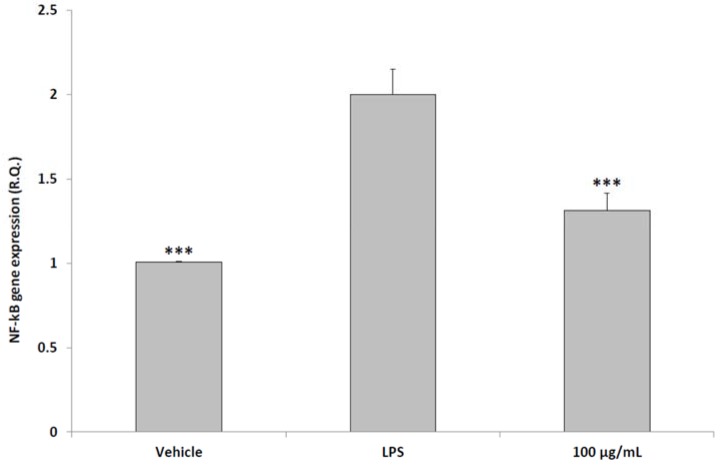
Effects of aqueous pollen extracts (100 μg/mL) on NFκB gene expression from isolated rat prostate specimens challenged with LPS (ANOVA, *p* < 0.0001; post-hoc test *** *p* < 0.001 vs. LPS group). Pollen extracts were given simultaneously with LPS.

The in vitro antioxidant effects induced by the pollen extract are consistent with the reported antiradical role of polyphenolic constituents of bee pollen [32,33]. On the other hand, it is not possible to exclude direct interactions between the treatments present in cell medium. The preliminary in vitro test revealed a valuable index of non-toxic and effective concentrations, which is useful to define the concentration for prostate tissue treatment. In a third set of experiments, we evaluated the modulatory effects using a aqueous pollen extract (100 μg/mL) supplementation on multiple inflammatory pathways, including PGE_2_ production and NFκB gene expression, in rat prostate specimens challenged with LPS. In this context, we evaluated the protective effect of pollen co-treated with LPS, compared to the inflammatory stimulus (LPS) alone. Additionally, in order to discriminate the inflammatory stimulus from the basal tissue levels of the tested inflammatory biomarkers, we included a negative control group constituted by the vehicle alone, without any treatment. As reported in multiple studies [9,34], ex vivo tissues challenged with LPS is a toxicity model for the evaluation of the efficacy of herbal extracts and drugs involved in inflammatory and oxidative stress modulation. Additionally, the stimulation of inflammatory and oxidative stress pathways in peripheral and central nervous system tissues has long been reported. For instance, lipid peroxidation and inflammatory biomarker production was described in brain synaptosomes, an ex vivo experimental model of brain tissues explants stimulated with drugs, herbal extracts and inflammatory stimuli soon after rat sacrifice [35,36]. Ex vivo tissues are sensitive to inflammatory stimuli until 4 h after sacrifice. We confirmed a protective effect induced by the extract that was able to blunt the increased PGE_2_ level and NFκB gene expression induced by LPS in prostate specimens (Figure 3 and Figure 4).

**Figure 5:**
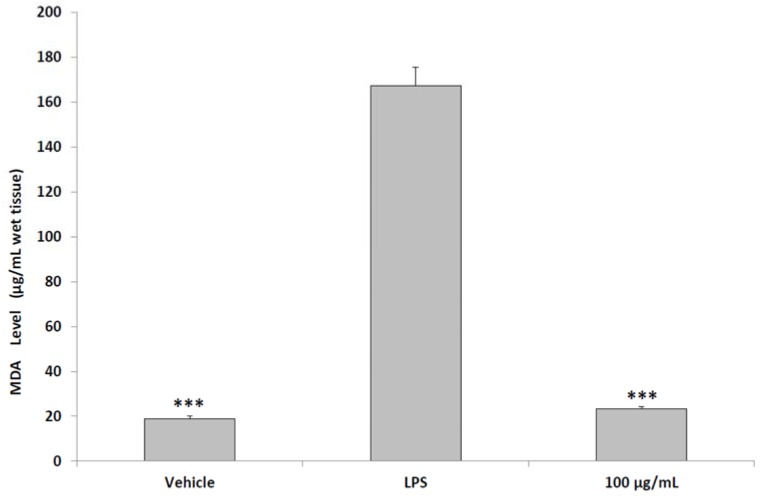
Effects of aqueous pollen extracts (100 μg/mL) on MDA production from isolated rat prostate specimens challenged with LPS (ANOVA, *p* < 0.0001; post-hoc test *** *p* < 0.001 vs. LPS group). Pollen extracts were given simultaneously with LPS.

The reported anti-inflammatory effects were consistent with previously published papers, that showed the capability of pollen to inhibit the production of IL-8 [5] and the activities of cyclooxygenase (COX)-2 and inducible nitric oxide synthase (iNOS), evaluated as a reduction of PGE_2_ and nitrites levels, respectively [37,38,39]. In the present work, together with the expression of NFκB, we also indirectly evaluated the activity of both enzymes. Actually, the inhibitory effect on NFκB gene expression could explain, albeit partially, this multitarget anti-inflammatory role. NFκB is a transcriptor factor involved in the expression of cytokines and inflammatory enzymes such as iNOS and COX-2 [40,41]. Previously, we reported the capability of a hydroalchoholic chamomile extract, containing a significant polyphenolic fraction, to simultaneously inhibit NFκB, PGE_2_, and nitrites pathways, in tissue specimens challenged with LPS [9]. The anti-inflammatory effects displayed by pollen ex vivo further confirm its efficacy for the prevention of prostatitis [4]. We also evaluated the effect of pollen supplementation (100 μg/mL) on lipid peroxidation, finding a significant reduction of MDA levels in pollen-treated group, compared to LPS group (Figure 5). MDA is a marker of lipid peroxidation mainly produced in the pathway NO-myeloperoxidase-MDA, in which NO levels were significantly increased during inflammatory process through the up-regulation of inducible iNOS [39].

Oxidative stress and lipid peroxidation have been related to structural changes in the prostate gland [42,43]. As previously reported [9], LPS stimulus was able to increase tissue lipid peroxidation ex vivo. The reduced MDA levels following pollen treatment were consistent with our findings of anti-oxidant and chelating properties displayed by pollen extract in vitro (Table 1). In order to highlight the presence in the tested agent of potentially active molecules, we investigated the phenolic composition of the pollen extract. A validated HPLC procedure was applied to identify and quantitate the amount of important secondary metabolites that could be responsible for the above reported biological activities, as previously demonstrated [44]. From the data in Table 2, reported as µg/g of pollen, this natural product was characterized by the high presence of specific secondary metabolites, including chlorogenic acid, gallic acid, rutin, quercetin, and carvacrol.

Rutin, carvacrol, and chlorogenic acid were reported to display inhibitory effects on NFκB activity [45,46,47]. On the other hand, all these metabolites could exert anti-oxidant and radical scavenger activities, further supporting the protective role of pollen extract supplementation [48,49,50,51]. Multiple clinical trials have also demonstrated the efficacy of bee pollen, administered orally, in blunting pain in patients suffering from chronic prostatitis [52,53]. On the other hand, the putative mechanism of action has not been completely investigated. Our observations indicated a multitarget action following pollen supplementation, with possible protective effects related to the reduction of multiple oxidative stress and inflammatory mediator levels, and to a significant cytotoxic effects on prostate cancer PC3 cell line, at the highest tested concentration (500 μg/mL). According to our experimental evidence, Graminex pollen appears to be a promising agent for the management of prostate inflammation. Finally, considering the relationship between prostatic inflammation and prostate cancer [54], we can also hypothesize a chemo-preventive effect induced by Graminex pollen as regards prostate cancer.

In addition, also if not biologically related to inflammation, we aimed at evaluating the pharmacological potential of this pollen against a panel of important target enzymes for the treatment of other human diseases. As reported in Table 3, this natural compound showed weak inhibitory activity against cholinesterases, α-amylase and α-glucosidase (inferior to standard drugs); however, discrete activity against tyrosinase with respect to kojic acid was observed.

Tyrosinase was evaluated in analogy with the recent study performed by Seo et al. (2018) [55], indicating the possible use of finasteride, the principle drug used in prostate diseases, as an inhibitor of melanogenesis, possibly related to its inhibition of tyrosinase activity. Additionally, a recent paper in press by Shoskes et al. (2018) [56] revealed a significant relationship between prostate dysfunctions and systemic conditions, including diabetes, cardiovascular and neurologic diseases. In this context, the inhibitory effects exerted on these enzymes including α-glucosidase, α-amylase and cholinesterases, could improve our knowledge of the possible clinical use of graminex pollen. Finally, regarding α-glucosidase activity, we investigated the effects of Graminex pollen on this enzyme, considering that Graminex pollen could potentially be used by patients suffering from prostate cancer who had undergone radiotherapy. In these patients, the inhibition of α-glucosidase could induce intestinal untoward reactions [57].

As previously described [58], pollen powder was divided into four different samples and subjected to colorimetric measurements. The four samples were then stored in airtight containers, placed at 55 ± 1 °C and analysed at 24, 72, 120, 144, 168, 216 and 288 h. The resulting mean values, completed by the standard deviation of the four analysed samples, are reported in Table 4.

**Figure 6:**
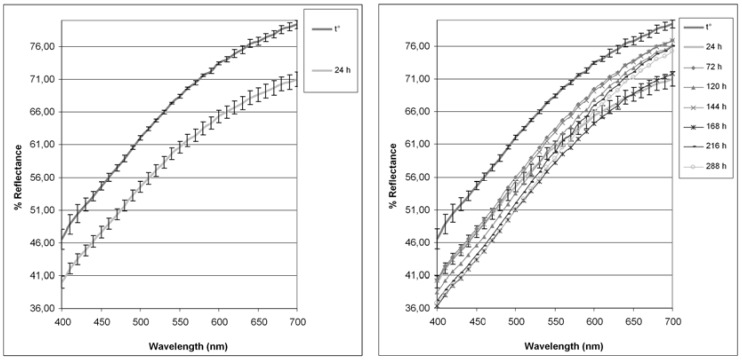
Spectral reflectance curves obtained by CIELAB colour analysis for Graminex pollen.

The samples showed a pale but dominant yellow colour, as demonstrated by the high value of L* (86.05), the low a* value (1.71), and the positive b* value (11.96), that both slightly darken in the first 24 h of storage at 55 °C, as shown by a decrease of L* and overlapping a* and b* values (81.14, 1.75, 12.05, respectively). The next measurements, as better demonstrated in Figure 6 where reflectance curves of the samples stored at 55 °C for up to twelve days (288 h) were reported, evidenced that, for times longer than 24 h, the browning process stopped (as well as demonstrated by the slopes between 600 and 700 nm), but that of red and yellow components rose, with a curve lowering in the region 400–550 nm (Figure 6).

To our knowledge, no reports exist about the colorimetric analysis of pollen, and only a few papers dealt about the colorimetric evaluation of honey [59,60]. Moreover, literature data only reported colorimetric parameters for solved samples analysed by spectrophotometry, and did not show any reflectance curves. For this reason, the interpretation of the real mean of these trends, that could involve more than a modification process, such as Maillard reaction or Strecker degradation, is difficult. It will be of interest to evaluate the occurrence, in more and different pollen samples, of carotenoids or other pigments, that could undergo bleaching rather than polymerization processes [61], in order to better understand these preliminary colorimetric analyses.

## 4. Conclusions

Herbal extracts characterized by known antioxidant and anti-inflammatory effects are under investigation for the efficacy in prostatitis treatment. The aim of the present work was to evaluate the protective effects of Graminex pollen in rat prostate specimens. We studied the role of pollen on multiple inflammatory pathways, including the reduction of PGE_2_, NFκB mRNA and malondialdehyde.

We also characterized, by means of chromatographic and colorimetric studies, the composition of Graminex pollen to better correlate the activity of pollen on immortalized prostate cells (PC3) and in rat prostate specimens challenged with *E. coli* lipopolysaccharide (LPS). We found that Graminex pollen was able to reduce ROS production by PC3 cells and MDA, NFκB mRNA, and PGE_2_ levels, in rat prostate specimens.

In conclusion, according to our experimental evidence, Graminex pollen appears to be a promising natural product for the management of the inflammatory components in the prostate. Considering the employed experimental model [25,42], our findings suggest protective effects of the Graminex pollen when applied before or with onset of the inflammation. Additionally, Graminex pollen was also revealed to be a promising enzyme inhibitory agent. However, the inhibitory effect on α-glucosidase would preclude its use in patients suffering from prostate cancer who had been subjected to radiotherapy.

## Figures and Tables

**Table 1:** Anti-oxidant and chelating activities of Graminex pollen.

Assays	Results *
Phosphomolybdenum (mg TEs/g)	32.07 ± 2.39
DPPH (mg TEs/g)	ni
ABTS (mg TEs/g)	8.55 ± 0.33
CUPRAC (mg TEs/g)	9.11 ± 0.76
FRAP (mg TEs/g)	6.95 ± 0.25
Metal Chelating (mg EDTAEs/g)	1.85 ± 0.03

* Values expressed are means ± S.D. of three experiments performed in triplicate (*p* < 0.05). TE: Trolox equivalent; EDTAE: EDTA equivalent. ni: no inhibition.

**Table 2:** Phenolic pattern of Graminex pollen.

Compound	µg/g of Pollen	Retention Time (min)	Wavelength (nm)
**Gallic acid**	89.06 ± 8.25	4.99	271
**Catechin**	nd	13.36	278
**Chlorogenic acid**	101.77 ± 10.09	14.29	324
***p*-OH benzoic acid**	nd	14.71	256
**Vanillic acid**	nd	17.31	260
**Epicatechin**	nd	18.30	278
**Syringic acid**	nd	18.50	274
**3-OH benzoic acid**	nd	19.41	275
**3-OH-4-MeO benzaldehyde**	nd	22.08	278
***p*-coumaric acid**	nd	22.65	310
**Rutin**	122.29 ± 11.23	25.38	256
**Sinapinic acid**	nd	26.18	324
***t*-ferulic acid**	nd	27.75	315
**Naringin**	nd	29.78	285
**2,3-diMeO benzoic acid**	nd	30.36	299
**Benzoic acid**	nd	31.20	275
***o*-coumaric acid**	nd	34.81	276
**Crocin**	nd	35.52	440
**Quercetin**	124.42 ± 12.01	40.57	367
**Harpagoside**	nd	45.49	280
***t*-cinnamic acid**	nd	45.87	276
**Naringenin**	nd	46.74	290
**Safranal**	nd	47.00	330
**Carvacrol**	251.88 ± 25.03	49.95	275
**TOTAL**	689.41 ± 52.89		

Data are reported as mean ± S.D. of three independent measurements. nd: not detected.

**Table 3:** Enzyme inhibition data for Graminex pollen.

Enzyme Inhibition	Result *
AChE Inhibition (mg GALAE/g)	0.26 ± 0.01
BChE Inhibition (mg GALAE/g)	0.35 ± 0.01
Tyrosinase inhibition (mg KAE/g)	1.53 ± 0.11
α-Amylase inhibition (mmol ACAE/g)	0.05 ± 0.01
α-Glucosidase inhibition (mmol ACAE/g)	0.79 ± 0.10

* Values expressed are means ± S.D. of three experiments performed in triplicate. GALAE: Galantamine equivalent; KAE: Kojic acid equivalent; ACAE: Acarbose equivalent.

**Table 4:** Color coordinates of Graminex pollen powder samples at the initial time and after storage at 55 °C.

	*t*°	24 h	72 h	120 h	144 h	168 h	216 h	288 h
***L***	86.05 ± 0.44	81.14 ± 0.39	83.39 ± 0.47	82.25 ± 0.31	83.14 ± 0.40	80.61 ± 0.20	81.63 ± 0.46	81.11 ± 0.70
a*	1.71 ± 0.11	1.75 ± 0.03	2.66 ± 0.11	3.24 ± 0.04	2.86 ± 0.14	2.92 ± 0.12	3.52 ± 0.08	3.69 ± 0.32
b*	11.96 ± 0.41	12.05 ± 0.15	14.30 ± 0.24	15.33 ± 0.34	14.48 ± 0.52	14.71 ± 0.41	15.60 ± 0.37	16.29 ± 1.14
C*_ab_	12.09 ± 0.39	12.18 ± 0.14	14.54 ± 0.25	15.67 ± 0.34	14.75 ± 0.54	15.00 ± 0.42	15.99 ± 0.37	16.05 ± 0.72
***h_ab_***	81.88 ± 0.79	81.78 ± 0.22	79.49 ± 0.31	78.05 ± 0.12	78.86 ± 0.13	78.80 ± 0.35	77.30 ±0.09	76.71 ± 0.56

The reported values are the means ± S.D. of four measurements.
